# Editorial Comment: Optimal timing of a second postoperative voiding trial in women with incomplete bladder emptying after vaginal reconstructive surgery: a randomized trial

**DOI:** 10.1590/S1677-5538.IBJU.2021.05.04

**Published:** 2021-07-23

**Authors:** Cássio L. Z. Riccetto

**Affiliations:** 1 Universidade Estadual de Campinas – UNICAMP Faculdade de Ciências Médicas Divisão de Urologia Feminina CampinasSP Brasil Divisão de Urologia Feminina, Faculdade de Ciências Médicas da Universidade Estadual de Campinas – UNICAMP, Campinas, SP, Brasil

Jeffrey S Schachar^1^, David Ossin^2^, Andre R Plair^1^, Eric A Hurtado^2^, Candace Parker-Autry^1^, Gopal Badlani^1^, et al.

Am J Obstet Gynecol. 2020 Aug;223(2):260.e1-260.e9.

## COMMENT

In this prospective randomized trial (RCT), the authors compared the results of a second attempt to remove the urethral catheter, performed 2-4 days versus 7 days postoperatively in women who had incomplete bladder emptying after surgical correction of vaginal prolapse involving at least 2 vaginal compartments. A hundred and two patients were initially enrolled in the study, 29 of which (28%) had voided normally - i.e. post-voiding residue less than 100 ml 6 hours after the procedure (with a bladder fully filled with 300 ml of saline and after the removal of the vaginal packing). After additional exclusions, 30 patients in each group were assessed using an intention to treat analysis. The authors concluded that women reevaluated in the 4th postoperative day were more likely to have inadequate urination (23.3%) compared to those reevaluated after 7 days (3.3%), resulting in a 20% risk difference [95% CI 3.56-36.44] and relative risk of 7.00 [95% CI 0.92-53.47], (p = 0.02). Severe postoperative pain and use of opioids were associated with higher rates of re-catheterization.

Surgical treatment of POP has become more frequent, especially in elderly patients. In this group, POP is often concomitant bladder dysfunction, which in turn makes the prediction of bladder function in early postoperative periods quite inaccurate. Moreover, there has been a trend towards shortening hospital stays and an increasing tendency towards outpatient POP procedures, in line to what already occurs for midurethral slings (1, 2). In our experience, patient attempts to urinate in the first postoperative period are often successful, with a low risk of high residual volume that would demand re-catheterization. Conversely, in this RCT, removal of the catheter after 6 hours of the procedure resulted in satisfactory voiding in only 28% of the patients at most, while extending catheterization to the fourth postoperative period would still result in incomplete voiding in approximately a quarter of patients. Such information would be very helpful to provide adequate preoperative counseling for our patients.

## References

[1] 1. Alas A, Martin L, Devakumar H, Frank L, Vaish S, Chandrasekaran N, Davila GW, Hurtado E. Anesthetics’ role in postoperative urinary retention after pelvic organ prolapse surgery with concomitant midurethral slings: a randomized clinical trial. Int Urogynecol J. 2020;31:205-13. 10.1007/s00192-019-03917-w 30904934

[2] 2. Yune JJ, Cheng JW, Wagner H, Kim J, Hardesty JS, Siddighi S. Postoperative urinary retention after pelvic organ prolapse repair: Vaginal versus robotic transabdominal approach. Neurourol Urodyn. 2018;37:1794-1800. 10.1002/nau.23526 29572921

